# Supplementary material for: Cost-Effectiveness Analysis of COVID-19 Vaccine Booster Dose in the Thai Setting during the Period of Omicron Variant Predominance
Source: Trop Med Infect Dis. 2023 Jan 30;8(2):91. doi: 10.3390/tropicalmed8020091 (PMC9959539; doi:10.3390/tropicalmed8020091)
Supplement: Supplementary file 1 [file tropicalmed-08-00091-s001.zip › Supp. File S2 model equation.pdf]

model d2p v13.stmx

Total	Count	Including Array Elements
Variables	173	326
Stocks	36	81
Flows	33	96
Converters	104	149
Constants	64	130
Equations	73	115
Graphicals	0	0

		Equation	Properties	Units	Documentation	Annotation
	Top-Level Model:					
	cum_vac_3_doses_additional_base(t)	cum_vac_3_doses_additional_base(t - dt) + (Flow_10) * dt	INIT cum_vac_3_doses_additional_base = 0			NON-NEGATIVE
	cum_vac_3_doses_additional_mRNA_scenario(t)	cum_vac_3_doses_additional_mRNA_scenario(t - dt) + (Flow_29) * dt	INIT cum_vac_3_doses_additional_mRNA_scenario = 0			NON-NEGATIVE
	cum_vac_3_doses_additional_vv_scenario(t)	cum_vac_3_doses_additional_vv_scenario(t - dt) + (Flow_12) * dt	INIT cum_vac_3_doses_additional_vv_scenario = 0			NON-NEGATIVE
	cumulative_reported_cases_cases[severity](t)	cumulative_reported_cases_cases[severity](t - dt) + (daily_reported_cases_by_severity[severity]) * dt	INIT cumulative_reported_cases_cases[severity] = 0			NON-NEGATIVE
	cumulative_reported_cases_cases_1[severity](t)	cumulative_reported_cases_cases_1[severity](t - dt) + (daily_reported_cases_by_severity_1[severity]) * dt	INIT cumulative_reported_cases_cases_1[severity] = 0			NON-NEGATIVE
	cumulative_reported_cases_cases_2[severity](t)	cumulative_reported_cases_cases_2[severity](t - dt) + (daily_reported_cases_by_severity_2[severity]) * dt	INIT cumulative_reported_cases_cases_2[severity] = 0			NON-NEGATIVE
	E(t)	E(t - dt) + (flow_1 - Flow_2[mild_asym] - Flow_2[pneumo_no_ETT] - Flow_2[pneumo_ETT_no_dead] - Flow_2[pneumo_ETT_dead]) * dt	INIT E = 0			NON-NEGATIVE
	E_1(t)	E_1(t - dt) + (Flow_5 - Flow_6[mild_asym] - Flow_6[pneumo_no_ETT] - Flow_6[pneumo_ETT_no_dead] - Flow_6[pneumo_ETT_dead]) * dt	INIT E_1 = 0			NON-NEGATIVE
	E_2(t)	E_2(t - dt) + (flow_11 - Flow_13[mild_asym] - Flow_13[pneumo_no_ETT] - Flow_13[pneumo_ETT_no_dead] - Flow_13[pneumo_ETT_dead]) * dt	INIT E_2 = 0			NON-NEGATIVE
	E_3(t)	E_3(t - dt) + (Flow_16 - Flow_17[mild_asym] - Flow_17[pneumo_no_ETT] - Flow_17[pneumo_ETT_no_dead] - Flow_17[pneumo_ETT_dead]) * dt	INIT E_3 = 0			NON-NEGATIVE
	E_4(t)	E_4(t - dt) + (flow_20 - Flow_22[mild_asym] - Flow_22[pneumo_no_ETT] - Flow_22[pneumo_ETT_no_dead] - Flow_22[pneumo_ETT_dead]) * dt	INIT E_4 = 0			NON-NEGATIVE
	E_5(t)	E_5(t - dt) + (Flow_25 - Flow_26[mild_asym] - Flow_26[pneumo_no_ETT] - Flow_26[pneumo_ETT_no_dead] - Flow_26[pneumo_ETT_dead]) * dt	INIT E_5 = 0			NON-NEGATIVE
	I_after[severity](t)	I_after[severity](t - dt) + (Flow_3[severity] - Flow_4[severity]) * dt	INIT I_after[severity] = 0<br>TRANSIT TIME = admission_time<br>CONTINUOUS<br>ACCEPT MULTIPLE BATCHES			CONVEYOR
	I_after_1[severity](t)	I_after_1[severity](t - dt) + (Flow_7[severity] - Flow_8[severity]) * dt	INIT I_after_1[severity] = 0<br>TRANSIT TIME = 1<br>CONTINUOUS<br>ACCEPT MULTIPLE BATCHES			CONVEYOR
	I_after_2[severity](t)	I_after_2[severity](t - dt) + (Flow_14[severity] - Flow_15[severity]) * dt	INIT I_after_2[severity] = 0<br>TRANSIT TIME = admission_time_1<br>CONTINUOUS<br>ACCEPT MULTIPLE BATCHES			CONVEYOR
	I_after_3[severity](t)	I_after_3[severity](t - dt) + (Flow_18[severity] - Flow_19[severity]) * dt	INIT I_after_3[severity] = 0<br>TRANSIT TIME = 1<br>CONTINUOUS<br>ACCEPT MULTIPLE BATCHES			CONVEYOR
	I_after_4[severity](t)	I_after_4[severity](t - dt) + (Flow_23[severity] - Flow_24[severity]) * dt	INIT I_after_4[severity] = 0<br>TRANSIT TIME = admission_time_2<br>CONTINUOUS<br>ACCEPT MULTIPLE BATCHES			CONVEYOR
	I_after_5[severity](t)	I_after_5[severity](t - dt) + (Flow_27[severity] - Flow_28[severity]) * dt	INIT I_after_5[severity] = 0<br>TRANSIT TIME = 1<br>CONTINUOUS<br>ACCEPT MULTIPLE BATCHES			CONVEYOR
	I_before[severity](t)	I_before[severity](t - dt) + (Flow_2[severity] - Flow_3[severity]) * dt	INIT I_before[severity] = total_pop*pct_infectious_all_starting*(1-pct_vac_3_doses_starting)*pct_severity_vac_less_than_3_doses<br>TRANSIT TIME = time_lag<br>CONTINUOUS<br>ACCEPT MULTIPLE BATCHES			CONVEYOR
	I_before_1[severity](t)	I_before_1[severity](t - dt) + (Flow_6[severity] - Flow_7[severity]) * dt	INIT I_before_1[severity] = total_pop*pct_severity_vac_3_more_doses*pct_infectious_all_starting*pct_vac_3_doses_starting<br>TRANSIT TIME = time_lag<br>CONTINUOUS<br>ACCEPT MULTIPLE BATCHES			CONVEYOR
	I_before_2[severity](t)	I_before_2[severity](t - dt) + (Flow_13[severity] - Flow_14[severity]) * dt	INIT I_before_2[severity] = total_pop_1*pct_infectious_all_starting_1*(1-pct_vac_3_doses_starting_1)*pct_severity_vac_less_than_3_doses_1<br>TRANSIT TIME = time_lag_1<br>CONTINUOUS<br>ACCEPT MULTIPLE BATCHES			CONVEYOR
	I_before_3[severity](t)	I_before_3[severity](t - dt) + (Flow_17[severity] - Flow_18[severity]) * dt	INIT I_before_3[severity] = total_pop_1*pct_severity_vac_3_more_doses_1*pct_infectious_all_starting_1*pct_vac_3_doses_starting_1<br>TRANSIT TIME = time_lag_1<br>CONTINUOUS<br>ACCEPT MULTIPLE BATCHES			CONVEYOR
	I_before_4[severity](t)	I_before_4[severity](t - dt) + (Flow_22[severity] - Flow_23[severity]) * dt	INIT I_before_4[severity] = total_pop_2*pct_infectious_all_starting_2*(1-pct_vac_3_doses_starting_2)*pct_severity_vac_less_than_3_doses_2<br>TRANSIT TIME = time_lag_2<br>CONTINUOUS<br>ACCEPT MULTIPLE BATCHES			CONVEYOR
	I_before_5[severity](t)	I_before_5[severity](t - dt) + (Flow_26[severity] - Flow_27[severity]) * dt	INIT I_before_5[severity] = total_pop_2*pct_severity_vac_3_more_doses_2*pct_infectious_all_starting_2*pct_vac_3_doses_starting_2<br>TRANSIT TIME = time_lag_2<br>CONTINUOUS<br>ACCEPT MULTIPLE BATCHES			CONVEYOR
	Removed(t)	Removed(t - dt) + (Flow_4[mild_asym] + Flow_4[pneumo_no_ETT] + Flow_4[pneumo_ETT_no_dead] + Flow_4[pneumo_ETT_dead]) * dt	INIT Removed = 0			NON-NEGATIVE
	Removed_1(t)	Removed_1(t - dt) + (Flow_8[mild_asym] + Flow_8[pneumo_no_ETT] + Flow_8[pneumo_ETT_no_dead] + Flow_8[pneumo_ETT_dead]) * dt	INIT Removed_1 = 0			NON-NEGATIVE
	Removed_2(t)	Removed_2(t - dt) + (Flow_15[mild_asym] + Flow_15[pneumo_no_ETT] + Flow_15[pneumo_ETT_no_dead] + Flow_15[pneumo_ETT_dead]) * dt	INIT Removed_2 = 0			NON-NEGATIVE
	Removed_3(t)	Removed_3(t - dt) + (Flow_19[mild_asym] + Flow_19[pneumo_no_ETT] + Flow_19[pneumo_ETT_no_dead] + Flow_19[pneumo_ETT_dead]) * dt	INIT Removed_3 = 0			NON-NEGATIVE
	Removed_4(t)	Removed_4(t - dt) + (Flow_24[mild_asym] + Flow_24[pneumo_no_ETT] + Flow_24[pneumo_ETT_no_dead] + Flow_24[pneumo_ETT_dead]) * dt	INIT Removed_4 = 0			NON-NEGATIVE
	Removed_5(t)	Removed_5(t - dt) + (Flow_28[mild_asym] + Flow_28[pneumo_no_ETT] + Flow_28[pneumo_ETT_no_dead] + Flow_28[pneumo_ETT_dead]) * dt	INIT Removed_5 = 0			NON-NEGATIVE
	S(t)	S(t - dt) + (- flow_1 - vac_rate) * dt	INIT S = Suscep_all_starting*(1-pct_vac_3_doses_starting)			NON-NEGATIVE
	S_1(t)	S_1(t - dt) + (vac_rate - Flow_5) * dt	INIT S_1 = Suscep_all_starting*pct_vac_3_doses_starting			NON-NEGATIVE
	S_2(t)	S_2(t - dt) + (- flow_11 - vac_rate_vv) * dt	INIT S_2 = Suscep_all_starting_1*(1-pct_vac_3_doses_starting_1)			NON-NEGATIVE
	S_3(t)	S_3(t - dt) + (vac_rate_vv - Flow_16) * dt	INIT S_3 = Suscep_all_starting_1*pct_vac_3_doses_starting_1			NON-NEGATIVE
	S_4(t)	S_4(t - dt) + (- flow_20 - vac_rate_mRNA) * dt	INIT S_4 = Suscep_all_starting_2*(1-pct_vac_3_doses_starting_2)			NON-NEGATIVE
	S_5(t)	S_5(t - dt) + (vac_rate_mRNA - Flow_25) * dt	INIT S_5 = Suscep_all_starting_2*pct_vac_3_doses_starting_2			NON-NEGATIVE
	daily_reported_cases_by_severity[mild_asym]	(Flow_3[mild_asym]+Flow_7[mild_asym])*underreporting_factor_mild_asym				UNIFLOW
	daily_reported_cases_by_severity[pneumo_no_ETT]	(Flow_3[pneumo_no_ETT]+Flow_7[pneumo_no_ETT])*underreporting_factor_pneumonia_no_ett				
	daily_reported_cases_by_severity[pneumo_ETT_no_dead]	(Flow_3[pneumo_ETT_no_dead]+Flow_7[pneumo_ETT_no_dead])*underreporting_factor_pneumonia_ett_NO_death				
	daily_reported_cases_by_severity[pneumo_ETT_dead]	(Flow_3[pneumo_ETT_dead]+Flow_7[pneumo_ETT_dead])*underreporting_factor_pneumonia_ett_death				
	daily_reported_cases_by_severity_1[mild_asym]	(Flow_14[mild_asym]+Flow_18[mild_asym])*underreporting_factor_mild_asym_1				UNIFLOW
	daily_reported_cases_by_severity_1[pneumo_no_ETT]	(Flow_14[pneumo_no_ETT]+Flow_18[pneumo_no_ETT])*underreporting_factor_pneumonia_no_ett_1				
	daily_reported_cases_by_severity_1[pneumo_ETT_no_dead]	(Flow_14[pneumo_ETT_no_dead]+Flow_18[pneumo_ETT_no_dead])*underreporting_factor_pneumonia_ett_NO_death_1				
	daily_reported_cases_by_severity_1[pneumo_ETT_dead]	(Flow_14[pneumo_ETT_dead]+Flow_18[pneumo_ETT_dead])*underreporting_factor_pneumonia_ett_death_1				
	daily_reported_cases_by_severity_2[mild_asym]	(Flow_23[mild_asym]+Flow_27[mild_asym])*underreporting_factor_mild_asym_2				UNIFLOW
	daily_reported_cases_by_severity_2[pneumo_no_ETT]	(Flow_23[pneumo_no_ETT]+Flow_27[pneumo_no_ETT])*underreporting_factor_pneumonia_no_ett_2				
	daily_reported_cases_by_severity_2[pneumo_ETT_no_dead]	(Flow_23[pneumo_ETT_no_dead]+Flow_27[pneumo_ETT_no_dead])*underreporting_factor_pneumonia_ett_NO_death_2				
	daily_reported_cases_by_severity_2[pneumo_ETT_dead]	(Flow_23[pneumo_ETT_dead]+Flow_27[pneumo_ETT_dead])*underreporting_factor_pneumonia_ett_death_2				
	flow_1	S*I_before_all*(reproduction_number/infectious_duration)*(1-"ve_<_3_doses")/total_pop	OUTFLOW PRIORITY: 1			UNIFLOW
	Flow_10	vac_rate				UNIFLOW
	flow_11	S_2*I_before_all_1*(reproduction_number_1/infectious_duration)*(1-"ve_<_3_doses_1")/total_pop_1	OUTFLOW PRIORITY: 1			UNIFLOW
	Flow_12	vac_rate_vv				UNIFLOW
	Flow_13[severity]	E_2*pct_severity_vac_less_than_3_doses_1/inc_period				UNIFLOW
	Flow_14[severity]	CONVEYOR OUTFLOW				
	Flow_15[severity]	CONVEYOR OUTFLOW				
	Flow_16	(1-"ve_>=_3_doses_1")*(reproduction_number_1/infectious_duration)*S_3*I_before_all_1/total_pop_1				UNIFLOW
	Flow_17[severity]	E_3*pct_severity_vac_3_more_doses_1/inc_period				UNIFLOW
	Flow_18[severity]	CONVEYOR OUTFLOW				

	Flow_19[severity]	CONVEYOR OUTFLOW				
	Flow_2[severity]	E*pct_severity_vac_less_than_3_doses/inc_period				UNIFLOW
	flow_20	S_4*I_before_all_2*(reproduction_number_2/infectious_duration)*(1-"ve_<_3_doses_2")/total_pop_2	OUTFLOW PRIORITY: 1			UNIFLOW
	Flow_22[severity]	E_4*pct_severity_vac_less_than_3_doses_2/inc_period				UNIFLOW
	Flow_23[severity]	CONVEYOR OUTFLOW				
	Flow_24[severity]	CONVEYOR OUTFLOW				
	Flow_25	(1-"ve_>=_3_doses_2")*(reproduction_number_2/infectious_duration)*S_5*I_before_all_2/total_pop_2				UNIFLOW
	Flow_26[severity]	E_5*pct_severity_vac_3_more_doses_2/inc_period				UNIFLOW
	Flow_27[severity]	CONVEYOR OUTFLOW				
	Flow_28[severity]	CONVEYOR OUTFLOW				
	Flow_29	vac_rate_mRNA				UNIFLOW
	Flow_3[severity]	CONVEYOR OUTFLOW				
	Flow_4[severity]	CONVEYOR OUTFLOW				
	Flow_5	(1-"ve_>=_3_doses")*(reproduction_number/infectious_duration)*S_1*I_before_all/total_pop				UNIFLOW
	Flow_6[severity]	E_1*pct_severity_vac_3_more_doses/inc_period				UNIFLOW
	Flow_7[severity]	CONVEYOR OUTFLOW				
	Flow_8[severity]	CONVEYOR OUTFLOW				
	vac_rate	0	OUTFLOW PRIORITY: 2			UNIFLOW
	vac_rate_mRNA	STEP(171300, 1)	OUTFLOW PRIORITY: 2			UNIFLOW
	vac_rate_vv	STEP(171300, 1)	OUTFLOW PRIORITY: 2			UNIFLOW
	admin_cost_and_storage	234				
	admin_cost_and_storage_1	234				
	admin_cost_and_storage_2	234				
	admission_time[mild_asym]	10				
	admission_time[pneumo_no_ETT]	14				
	admission_time[pneumo_ETT_no_dead]	21				
	admission_time[pneumo_ETT_dead]	21				
	admission_time_1[mild_asym]	10				
	admission_time_1[pneumo_no_ETT]	14				
	admission_time_1[pneumo_ETT_no_dead]	21				
	admission_time_1[pneumo_ETT_dead]	21				
	admission_time_2[mild_asym]	10				
	admission_time_2[pneumo_no_ETT]	14				
	admission_time_2[pneumo_ETT_no_dead]	21				
	admission_time_2[pneumo_ETT_dead]	21				
	cum_ALL_cases_base	SUM(cumulative_reported_cases_cases)				
	cum_ALL_cases_mRNA	SUM(cumulative_reported_cases_cases_2)				
	cum_ALL_cases_vv	SUM(cumulative_reported_cases_cases_1)				
	cum_Death_base	cumulative_reported_cases_cases[pneumo_ETT_dead]				
	cum_Death_mRNA	cumulative_reported_cases_cases_2[pneumo_ETT_dead]				
	cum_Death_vv	cumulative_reported_cases_cases_1[pneumo_ETT_dead]				
	cum_Rx_cost_cases_base	SUM(total_Rx_cost_by_severity)				
	cum_Rx_cost_cases_mRNA	SUM(total_Rx_cost_by_severity_2)				
	cum_Rx_cost_cases_vv	SUM(total_Rx_cost_by_severity_1)				
	cum_Rx_cost_death_base	total_Rx_cost_by_severity[pneumo_ETT_dead]*cum_Death_base				
	cum_Rx_cost_death_mRNA	total_Rx_cost_by_severity_2[pneumo_ETT_dead]*cum_Death_mRNA				
	cum_Rx_cost_death_vv	total_Rx_cost_by_severity_1[pneumo_ETT_dead]*cum_Death_vv				
	daily_death	daily_reported_cases_by_severity[pneumo_ETT_dead]				
	daily_death_1	daily_reported_cases_by_severity_1[pneumo_ETT_dead]				
	daily_death_2	daily_reported_cases_by_severity_2[pneumo_ETT_dead]				
	daily_reported_cases_ALL_base	SUM(daily_reported_cases_by_severity)				
	daily_reported_cases_ALL_mRNA	SUM(daily_reported_cases_by_severity_2)				
	daily_reported_cases_ALL_vv	SUM(daily_reported_cases_by_severity_1)				
	I_before_all	I_before[mild_asym] + I_before_1[mild_asym]				SUMMING CONVERTER
	I_before_all_1	I_before_2[mild_asym] + I_before_3[mild_asym]				SUMMING CONVERTER
	I_before_all_2	I_before_4[mild_asym] + I_before_5[mild_asym]				SUMMING CONVERTER
	ICER_total_cost_per_cases_saved_BASE_vs_mRNA	IF TIME < 2 THEN 0 ELSE "incremental_total_cost_(mRNA-basE)"/"incremental_total_cases_saved_(base_-_mRNA)"				
	ICER_total_cost_per_cases_saved_BASE_vs_VV	IF TIME < 2 THEN 0 ELSE "incremental_total_cost_(vv-basE)"/"incremental_total_cases_saved_(base_-_vv)"				
	ICER_total_cost_per_death_saved_BASE_vs_mRNA	IF TIME < 2 THEN 0 ELSE "incremental_total_cost_(mRNA-basE)"/"incremental_total_death_saved_(base_-_mRNA)"				
	ICER_total_cost_per_death_saved_BASE_vs_VV	IF TIME < 2 THEN 0 ELSE "incremental_total_cost_(vv-basE)"/"incremental_total_death_saved_(base-vv)"				
	ICER_vac_cost_per_cases_save_BASE_vs_mRNA	IF TIME <2 THEN 0 ELSE margin_vac_3_cost_mRNA/"incremental_total_cases_saved_(base_-_mRNA)"				
	ICER_vac_cost_per_cases_save_BASE_vs_VV	IF TIME < 2 THEN 0 ELSE margin_vac_3_cost_vv/"incremental_total_cases_saved_(base_-_vv)"				
	ICER_vac_cost_per_death_save_BASE_vs_mRNA	IF TIME < 2 THEN 0 ELSE margin_vac_3_cost_mRNA/"incremental_total_death_saved_(base_-_mRNA)"				
	ICER_vac_cost_per_death_save_BASE_vs_VV	IF TIME < 2 THEN 0 ELSE margin_vac_3_cost_vv/"incremental_total_death_saved_(base-vv)"				
	inc_period	3.5				
	"incremental_total_cases_saved_(base_-_mRNA)"	cum_ALL_cases_base-cum_ALL_cases_mRNA				
	"incremental_total_cases_saved_(base_-_vv)"	cum_ALL_cases_base-cum_ALL_cases_vv				
	"incremental_total_cost_(mRNA-basE)"	cum_Rx_cost_cases_mRNA+total_third_dose_vac_mRNA-(cum_Rx_cost_cases_base+total_third_dose_vac_cost_BASE)				
	"incremental_total_cost_(vv-basE)"	total_third_dose_vac_cost_vv+cum_Rx_cost_cases_vv-(cum_Rx_cost_cases_base+total_third_dose_vac_cost_BASE)				
	"incremental_total_death_saved_(base_-_mRNA)"	cum_Death_base-cum_Death_mRNA				
	"incremental_total_death_saved_(base-vv)"	cum_Death_base-cum_Death_vv				
	infectious_duration	4.7				
	logOR_base	-0.580732				
	logOR_mRNA	-0.798307				
	logOR_vv	-0.417149				
	margin_cum_vac_3_doses_mRNA_base	cum_vac_3_doses_additional_mRNA_scenario-cum_vac_3_doses_additional_base				
	margin_cum_vac_3_doses_vv_base	cum_vac_3_doses_additional_vv_scenario-cum_vac_3_doses_additional_base				
	margin_vac_3_cost_mRNA	margin_cum_vac_3_doses_mRNA_base*(vial_cost_mRNA+admin_cost_and_storage_2)				
	margin_vac_3_cost_vv	margin_cum_vac_3_doses_vv_base*(admin_cost_and_storage_1+vial_cost_vv)				
	pct_infectious_all_starting	0.0025				
	pct_infectious_all_starting_1	0.0025				
	pct_infectious_all_starting_2	0.0025				
	pct_severity_vac_3_more_doses[mild_asym]	0.999487				
	pct_severity_vac_3_more_doses[pneumo_no_ETT]	0.000466				
	pct_severity_vac_3_more_doses[pneumo_ETT_no_dead]	0.0000155				
	pct_severity_vac_3_more_doses[pneumo_ETT_dead]	0.000031				
	pct_severity_vac_3_more_doses_1[mild_asym]	0.999487+STEP(0.0001890912, 78)				
	pct_severity_vac_3_more_doses_1[pneumo_no_ETT]	0.000466+STEP(-0.0001718427, 78)				
	pct_severity_vac_3_more_doses_1[pneumo_ETT_no_dead]	0.0000155+STEP(-0.0000057, 78)				
	pct_severity_vac_3_more_doses_1[pneumo_ETT_dead]	0.000031+STEP(-0.00001145, 78)				
	pct_severity_vac_3_more_doses_2[mild_asym]	0.999487+STEP(-0.000081, 78)				
	pct_severity_vac_3_more_doses_2[pneumo_no_ETT]	0.000466+STEP(0.00007368, 78)				
	pct_severity_vac_3_more_doses_2[pneumo_ETT_no_dead]	0.0000155+STEP(0.0000024547, 78)				
	pct_severity_vac_3_more_doses_2[pneumo_ETT_dead]	0.000031+STEP(0.0000049, 78)				
	pct_severity_vac_less_than_3_doses[mild_asym]	0.99729				
	pct_severity_vac_less_than_3_doses[pneumo_no_ETT]	0.002463				
	pct_severity_vac_less_than_3_doses[pneumo_ETT_no_dead]	0.000082				
	pct_severity_vac_less_than_3_doses[pneumo_ETT_dead]	0.000164				
	pct_severity_vac_less_than_3_doses_1[mild_asym]	0.99729				
	pct_severity_vac_less_than_3_doses_1[pneumo_no_ETT]	0.002463				
	pct_severity_vac_less_than_3_doses_1[pneumo_ETT_no_dead]	0.000082				
	pct_severity_vac_less_than_3_doses_1[pneumo_ETT_dead]	0.000164				
	pct_severity_vac_less_than_3_doses_2[mild_asym]	0.99729				
	pct_severity_vac_less_than_3_doses_2[pneumo_no_ETT]	0.002463				
	pct_severity_vac_less_than_3_doses_2[pneumo_ETT_no_dead]	0.000082				
	pct_severity_vac_less_than_3_doses_2[pneumo_ETT_dead]	0.000164				
	pct_vac_3_doses_starting	0				
	pct_vac_3_doses_starting_1	0.2				
	pct_vac_3_doses_starting_2	0.2				
	reproduction_number	1.5				
	reproduction_number_1	1.7				
	reproduction_number_2	1.7				
	Suscep_all_starting	total_pop*(1-pct_infectious_all_starting)				
	Suscep_all_starting_1	total_pop_1*(1-pct_infectious_all_starting_1)				
	Suscep_all_starting_2	total_pop_2*(1-pct_infectious_all_starting_2)				

	time_lag	4				
	time_lag_1	4				
	time_lag_2	4				
	total_pop	50*10^6				
	total_pop_1	50*10^6				
	total_pop_2	50*10^6				
	total_Rx_cost_by_severity[severity]	unit_cost_for_each_level_of_care*cumulative_reported_cases_cases				
	total_Rx_cost_by_severity_1[severity]	unit_cost_for_each_level_of_care_1*cumulative_reported_cases_cases_1				
	total_Rx_cost_by_severity_2[severity]	unit_cost_for_each_level_of_care_2*cumulative_reported_cases_cases_2				
	total_third_dose_vac_cost_BASE	(admin_cost_and_storage+vial_cost_base)*cum_vac_3_doses_additional_base				
	total_third_dose_vac_cost_vv	total_third_dose_vac_cost_BASE+margin_vac_3_cost_vv				
	total_third_dose_vac_mRNA	total_third_dose_vac_cost_BASE+margin_vac_3_cost_mRNA				
	underreporting_factor_mild_asym	0.16667				
	underreporting_factor_mild_asym_1	0.16667				
	underreporting_factor_mild_asym_2	0.16667				
	underreporting_factor_pneumonia_ett_death	1				
	underreporting_factor_pneumonia_ett_death_1	1				
	underreporting_factor_pneumonia_ett_death_2	1				
	underreporting_factor_pneumonia_ett_NO_death	1				
	underreporting_factor_pneumonia_ett_NO_death_1	1				
	underreporting_factor_pneumonia_ett_NO_death_2	1				
	underreporting_factor_pneumonia_no_ett	0.33333				
	underreporting_factor_pneumonia_no_ett_1	0.33333				
	underreporting_factor_pneumonia_no_ett_2	0.33333				
	unit_cost_for_each_level_of_care[mild_asym]	23000				
	unit_cost_for_each_level_of_care[pneumo_no_ETT]	81000				
	unit_cost_for_each_level_of_care[pneumo_ETT_no_dead]	252000				
	unit_cost_for_each_level_of_care[pneumo_ETT_dead]	252000				
	unit_cost_for_each_level_of_care_1[mild_asym]	23000				
	unit_cost_for_each_level_of_care_1[pneumo_no_ETT]	81000				
	unit_cost_for_each_level_of_care_1[pneumo_ETT_no_dead]	252000				
	unit_cost_for_each_level_of_care_1[pneumo_ETT_dead]	252000				
	unit_cost_for_each_level_of_care_2[mild_asym]	23000				
	unit_cost_for_each_level_of_care_2[pneumo_no_ETT]	81000				
	unit_cost_for_each_level_of_care_2[pneumo_ETT_no_dead]	252000				
	unit_cost_for_each_level_of_care_2[pneumo_ETT_dead]	252000				
	"ve_<_3_doses"	0				
	"ve_<_3_doses_1"	0				
	"ve_<_3_doses_2"	0				
	"ve_>=_3_doses"	1-EXP(logOR_base)				
	"ve_>=_3_doses_1"	IF TIME < 78 THEN 1-EXP(logOR_base) ELSE 1-EXP(logOR_vv)				
	"ve_>=_3_doses_2"	IF TIME < 78 THEN 1-EXP(logOR_base) ELSE 1-EXP(logOR_mRNA)				
	vial_cost_base	457				
	vial_cost_mRNA	488				
	vial_cost_vv	308				

Run Specs	
Start Time	1
Stop Time	170
DT	1/4
Fractional DT	True
Save Interval	1
Sim Duration	1.5
Time Units	Day
Pause Interval	0
Integration Method	Euler
Keep all variable results	True
Run By	Run
Calculate loop dominance information	True
Exhaustive Search Threshold	1000

Array Dimension	Indexed by	Elements
severity	Label (4)	mild_asym<br>pneumo_no_ETT<br>pneumo_ETT_no_dead<br>pneumo_ETT_dead
