# Supplementary figures and images for: Cost-Effectiveness Analysis of COVID-19 Vaccine Booster Dose in the Thai Setting during the Period of Omicron Variant Predominance
Source: Trop Med Infect Dis. 2023 Jan 30;8(2):91. doi: 10.3390/tropicalmed8020091 (PMC9959539; doi:10.3390/tropicalmed8020091)

## BASE

< 3 vacs

>= 3 vacs

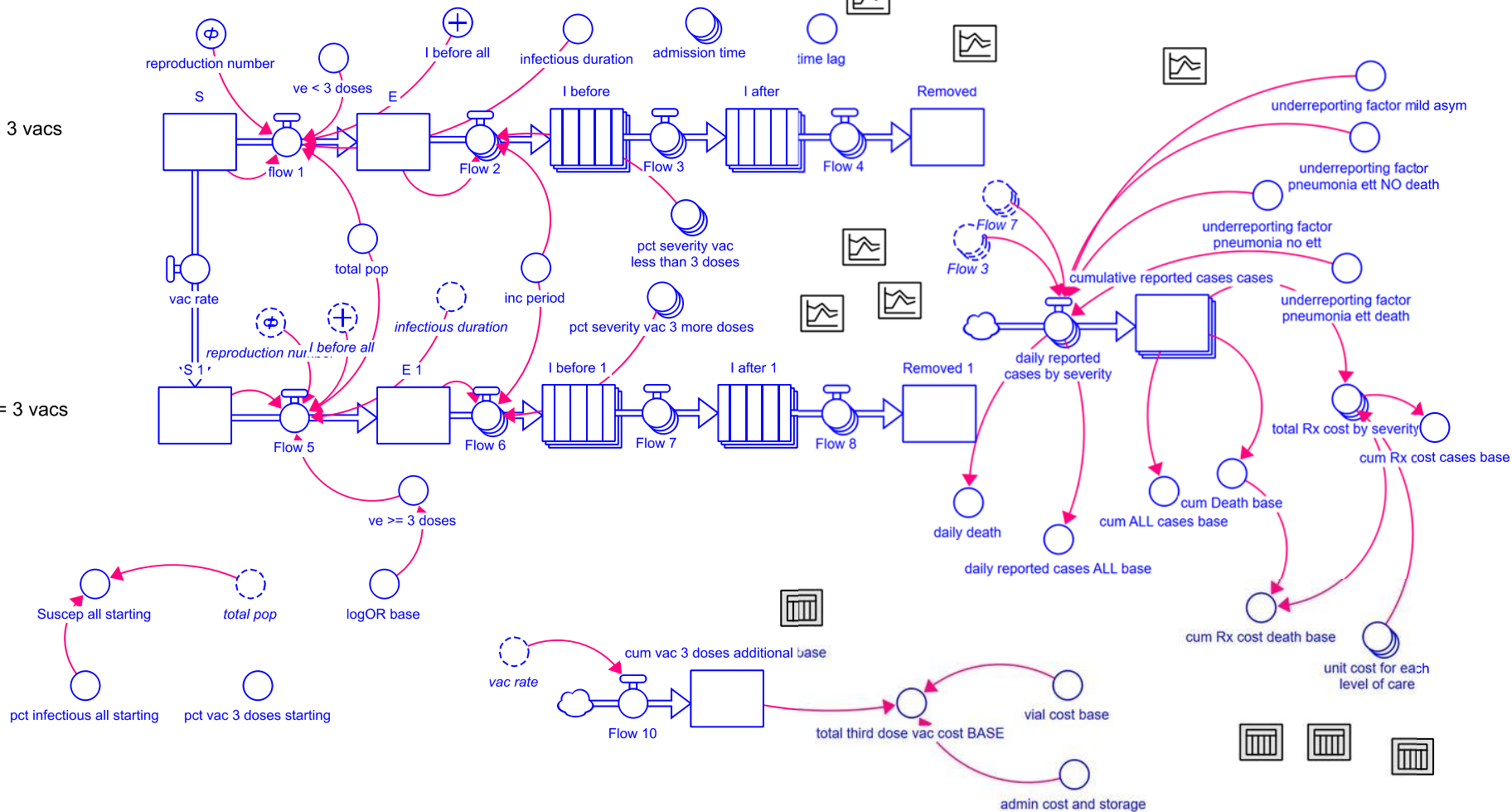

## Viral vector

< 3 vacs

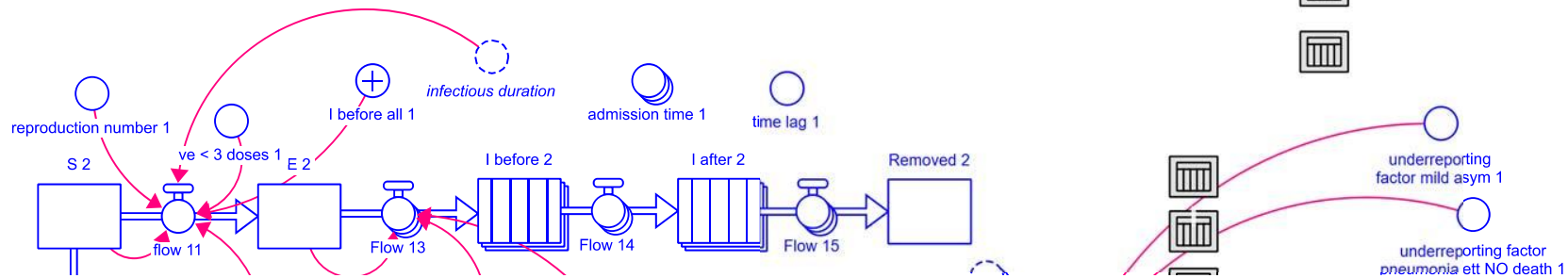

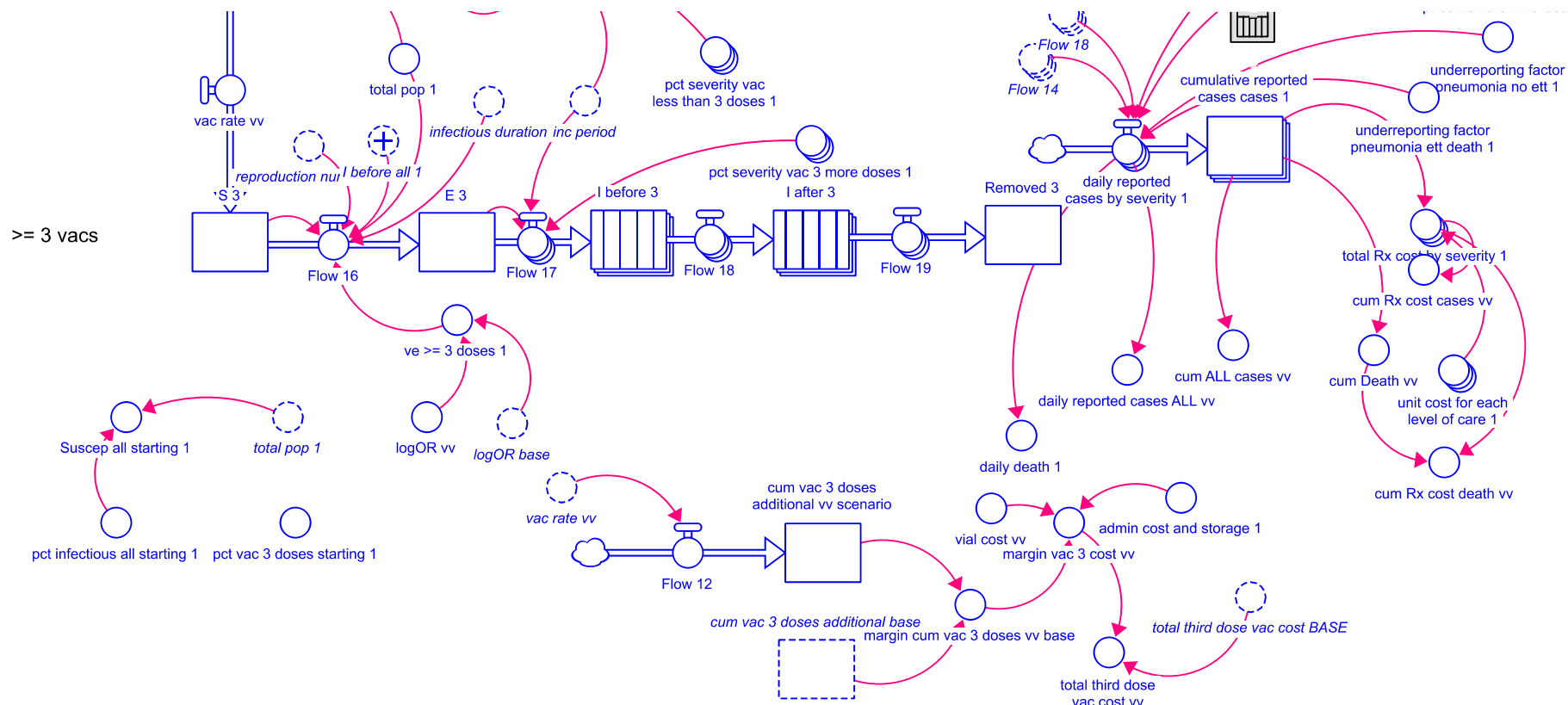

mRNA

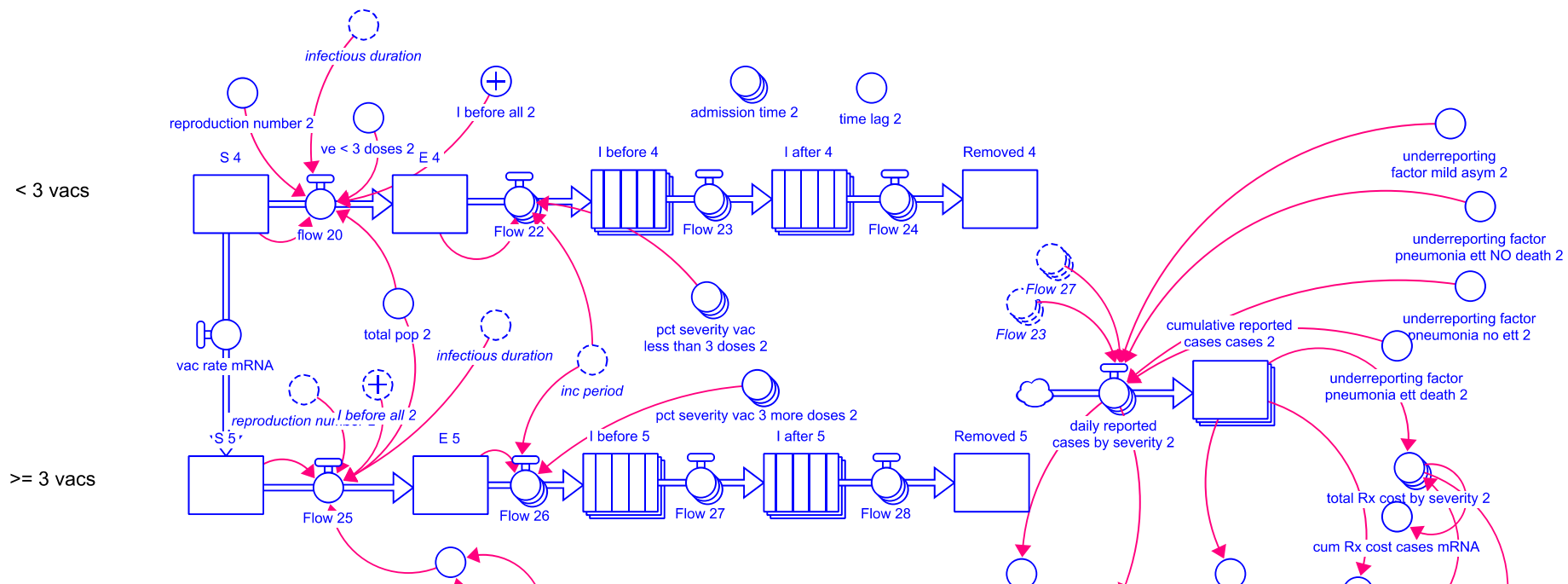

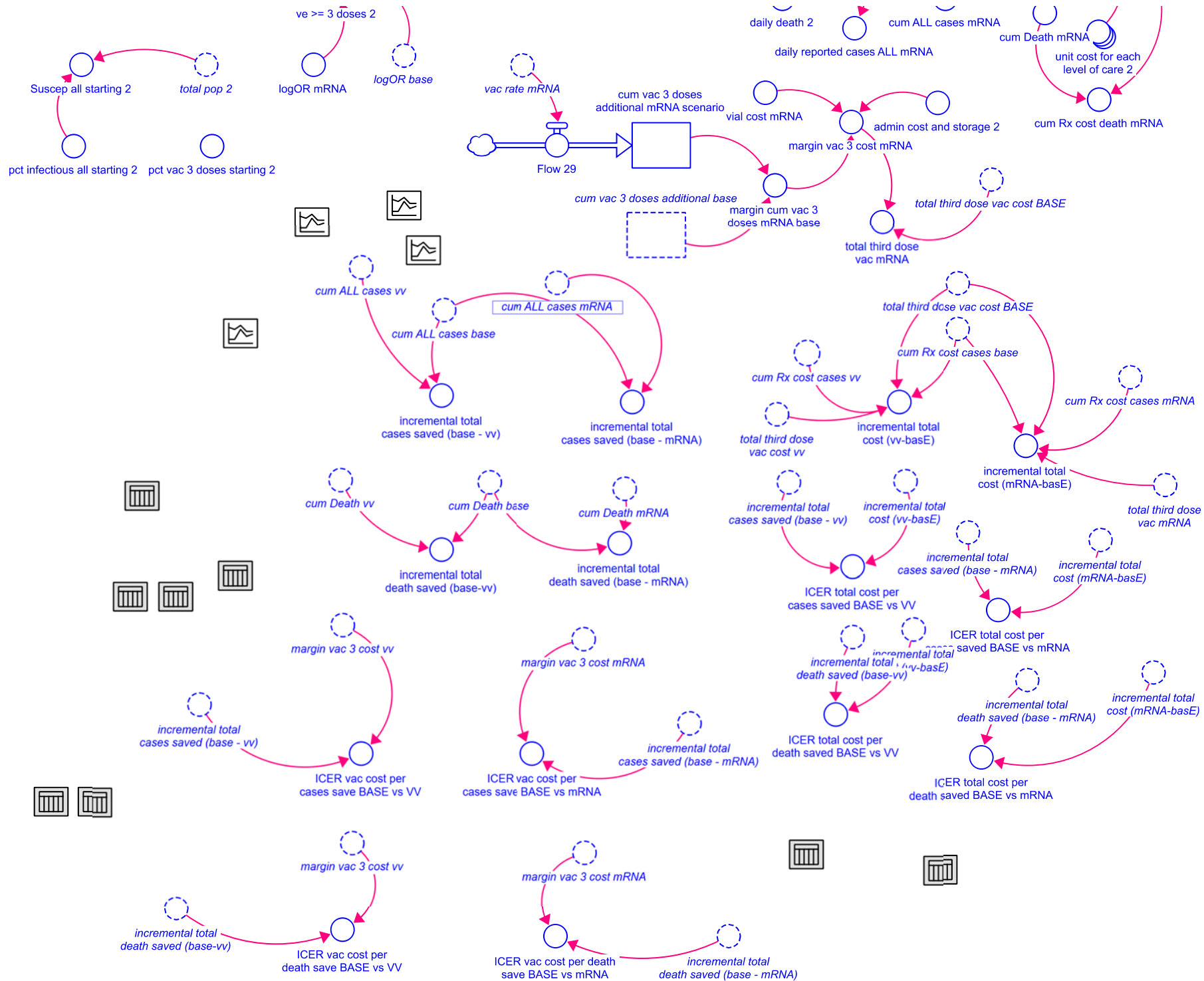

Supplement: Supplementary file 1 [file tropicalmed-08-00091-s001.zip › Supp. File S1 Model diagram.pdf]
